# Endoscopic treatments prior to urethroplasty: trends in management of urethral stricture disease

**DOI:** 10.1186/s12894-020-00638-x

**Published:** 2020-06-13

**Authors:** Matthew J. Moynihan, Bryan Voelzke, Jeremy Myers, Benjamin N. Breyer, Bradley Erickson, Sean P. Elliott, Nejd Alsikafi, Jill Buckley, Lee Zhao, Thomas Smith, Alex J. Vanni

**Affiliations:** 1 Department of Urology, Lahey Hospital and Medical Center, 41 Mall Rd, Burlington, MA 01805 USA; 2 Spokane Urology, Spokane, Washington USA; 3 University of Utah, Salt Lake City, UT USA; 4 University of California – San Francisco, San Francisco, California USA; 5 University of Iowa, Iowa City, Iowa USA; 6 University of Minnesota, Minneapolis, MN USA; 7 Uropartners, Gurnee, IL USA; 8 University of California – San Diego, San Diego, California USA; 9 New York University, Langone Medical Center, New York City, New York USA; 10 Baylor College of Medicine, Houston, TX USA

**Keywords:** Urethroplasty, Urethral stricture guidelines, Endoscopic treatment, Urethral stricture

## Abstract

**Background:**

To determine if the number of endoscopic treatments of urethral stricture disease (USD) prior to urethroplasty has changed in the context of new AUA guidelines on management of USD. In addition to an increase in practicing reconstructive urologists and published reconstructive literature, the AUA guidelines regarding the management of male USD were presented in May 2016, advocating consideration of urethroplasty in patients with 1 prior failed endoscopic treatment.

**Methods:**

A retrospective review of a prospectively maintained, multi-institutional urethral stricture database of high volume, geographically diverse institutions was performed from 2006 to 2017. We performed a review of relevant literature and evaluated pre-urethroplasty endoscopic treatment patterns prior to and after the AUA male stricture guideline.

**Results:**

2964 urethroplasties were reviewed in 10 institutions. There was both a decrease in the number of endoscopic treatments prior to urethroplasty in the pre-May 2016 compared to post-May 2016 cohorts both for overall urethroplasties (2.3 vs 1.6, *P* = 0.0012) and a gradual decrease in the number of pre-urethroplasty endoscopic treatments over the entire study period.

**Conclusion:**

There was a decrease in the number of endoscopic treatments of USD prior to urethroplasty in the observed period of interest. Declining endoscopic USD management is not likely to be a reflection of a solely unique influence of the guidelines as endoscopic treatment decreased over the entire study period. Further research is needed to determine if there will be a continued trend in the declining use of endoscopic treatment and elucidate the barriers to earlier urethroplasty in patients with USD.

## Background

Urologists play a critical role in the evaluation and management of urethral stricture disease (USD), which accounts for nearly 1.5 million office visits per year [1]. This condition has most commonly been managed with endoscopic treatments such as urethral dilation and direct vision internal urethrotomy (DVIU) [2]. However, endoscopic management has poor success with stricture recurrence rates between 50 and 92% [3, 4]. Additionally, multiple failed endoscopic treatments can make subsequent urethral reconstruction more challenging [5–7]. As such, a shift in management towards earlier urethroplasty has been seen in recent years, which has higher success rates and better reported patient outcomes [8].

Unfortunately, consensus on the management of male USD has historically been hindered by a lack of definitive practice recommendations. In fact, despite the available evidence, a retrospective single institutional review by Granieri and Peterson saw no measurable change in practice patterns before referral to urethroplasty from 1996 to 2010 [9]. In order to more definitively set recommendations for the urologic community, the American Urological Association (AUA) published its first set of evidence based guidelines regarding male urethral stricture disease in 2016 [10]. The guidelines are notable for their recommendations for earlier urethroplasty and avoidance of repeated endoscopic management for recurrent urethral strictures. Specifically, the AUA male urethral stricture guideline recommends that patients should be offered urethroplasty after failed endoscopic management (Guideline 11) and also for fossa navicularis, penile, bulbar strictures > 2 cm, and recurrent bulbar strictures (Guidelines 13–16) [10]. However, it is unclear if the new guideline has decreased the number of endoscopic treatments prior to referral for urethroplasty. Our objective is to determine if there has been a decrease in the use of endoscopic treatment of USD in high volume reconstructive practices over time and whether the AUA guideline has accelerated this impact. We hypothesize that the number of endoscopic treatments prior to urethroplasty in the cohort has decreased over time and that the guideline has further decreased the use of endoscopic treatment prior to referral.

## Methods

Institutional review board approval was obtained for this study (Lahey Hospital and Medical Center Institutional Review Board #20193211). We performed a literature review of publications describing factors driving changes in the utilization of urethroplasty. We then conducted a retrospective review of a prospectively maintained multi-institutional database of all patients who underwent a urethroplasty performed by a total of 10 surgeons between years 2006–2017. Patient demographics, length of stricture, etiology, and pre-operative endoscopic interventions were recorded. Either DVIU or urethral dilation were considered to be endoscopic treatment. To determine if pre-urethroplasty endoscopic treatment patterns prior to referral changed after the May 2016 AUA stricture guidelines, the number of endoscopic treatments prior to urethroplasty were recorded and grouped into pre-May 2016 and post-May 2016 cohorts. Statistics were performed with Chi-square tests and t-tests where appropriate.

## Results

A total of 2964 urethroplasties performed by 10 surgeons were reviewed that had sufficient data for analysis. Patient demographics can be found in Table 1. Those undergoing posterior urethroplasty were statistically significantly more likely to be older, hypertensive, hyperlipidemic, have a history of malignancy, and history of pelvic radiation (all *P* < 0.0001).

**Table 1:**
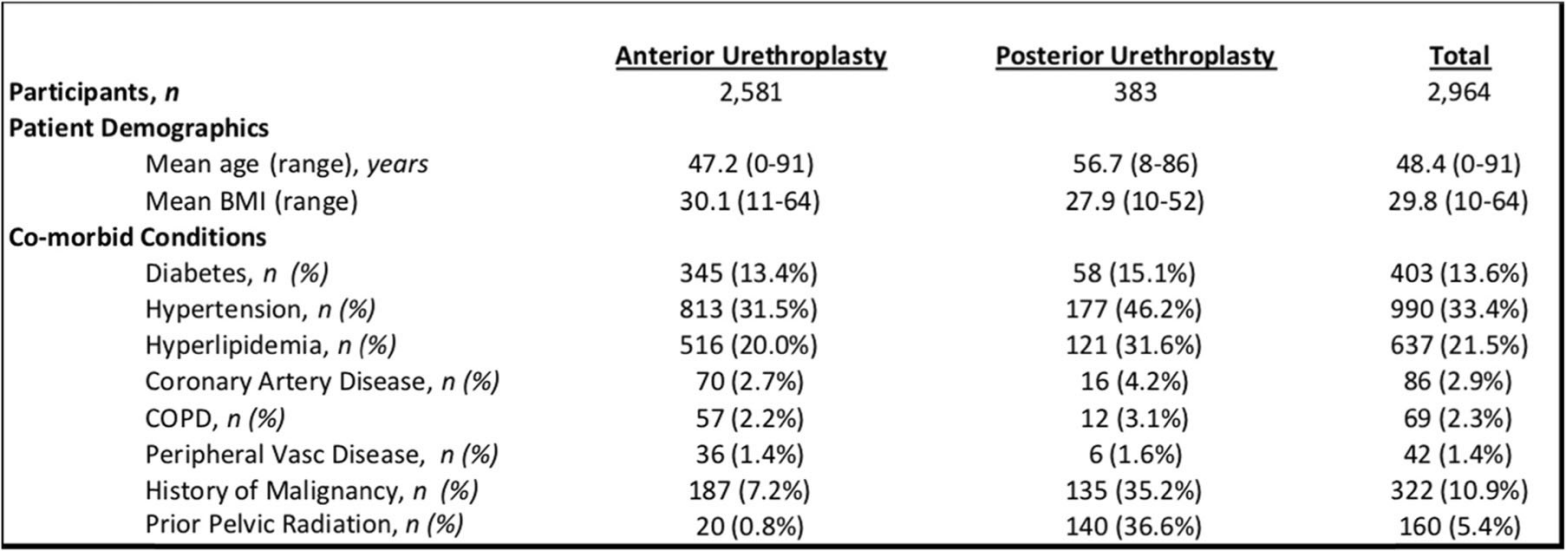
Patient demographics and characteristics

There was no difference in overall mean stricture length between cohorts (3.8 cm vs 3.7 cm, *P* = 0.5928). Looking specifically at etiology of anterior urethroplasties, idiopathic strictures were the most common etiology (43%). The pre-May 2016 cohort had a greater proportion of traumatic (15.2% vs 10.5%, *P* = 0.0022) strictures. The post-May 2016 cohort had a greater proportion of anterior urethroplasties performed for iatrogenic (22.0% vs 16.2%, *P* = 0.0006) and failed hypospadias (9.76% vs 6.3%, *P* = 0.0027) etiologies. There was no significant difference in proportion of idiopathic, infectious, lichen sclerosus, or radiation as the etiology of the anterior USD between the two cohorts (Table 2).

**Table 2:**
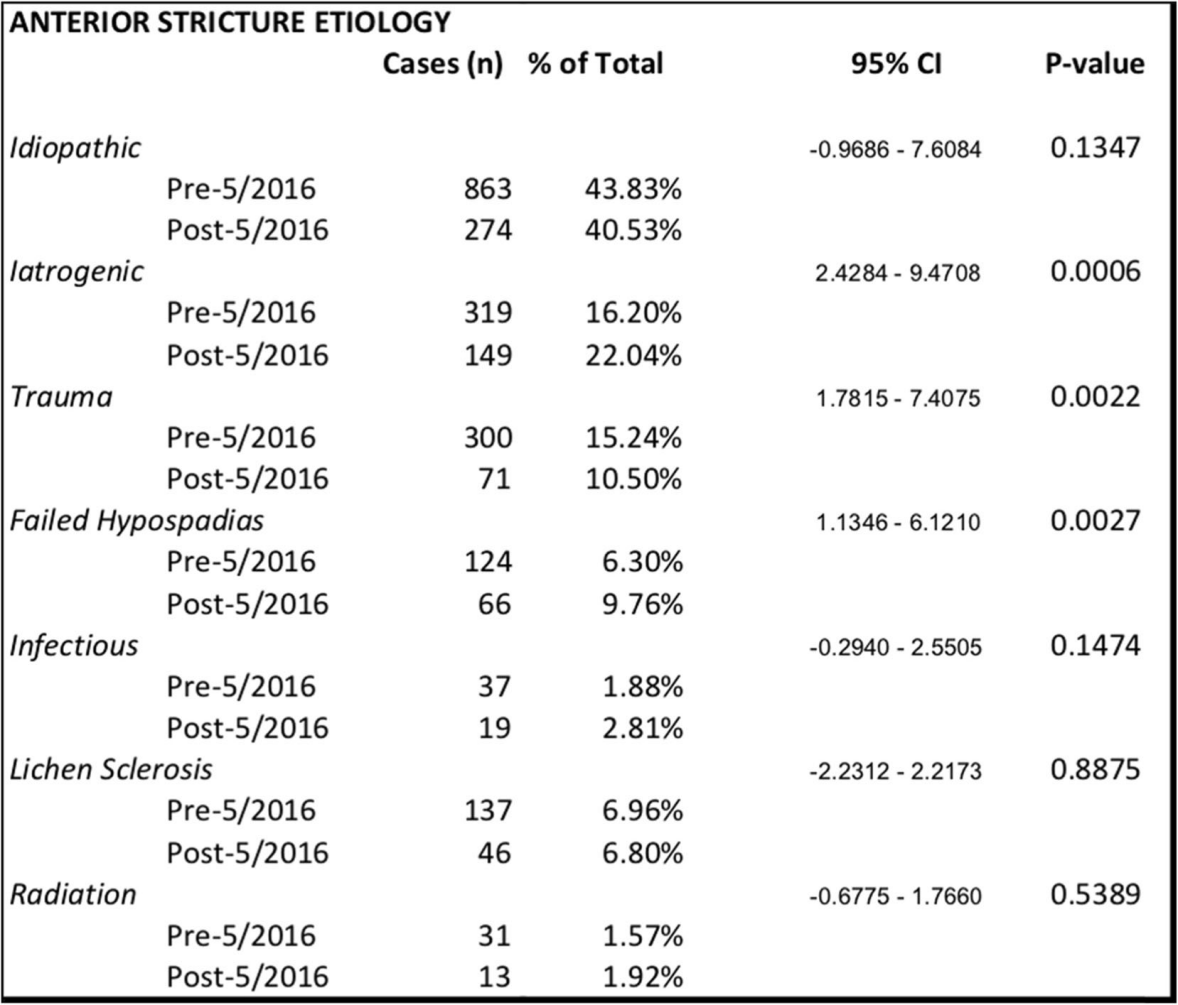
Etiology of anterior urethral stricture disease by cohort with respect to release of AUA urethral stricture disease guidelines

Overall average number of endoscopic treatments prior to urethroplasty for the entire cohort was 2.15 (SD = 4.887). Endoscopic treatment prior to urethroplasty is less common in patients undergoing posterior urethral reconstruction compared to anterior (0.38 vs 2.0, *P* < 0.0001). With regard to anterior urethroplasties, a majority of patients (69.8%) had at least one endoscopic treatment prior to urethroplasty. For all urethroplasties, 37.7% had at least 2 endoscopic treatments (range 0–91) prior to undergoing urethroplasty.

**Fig. 1:**
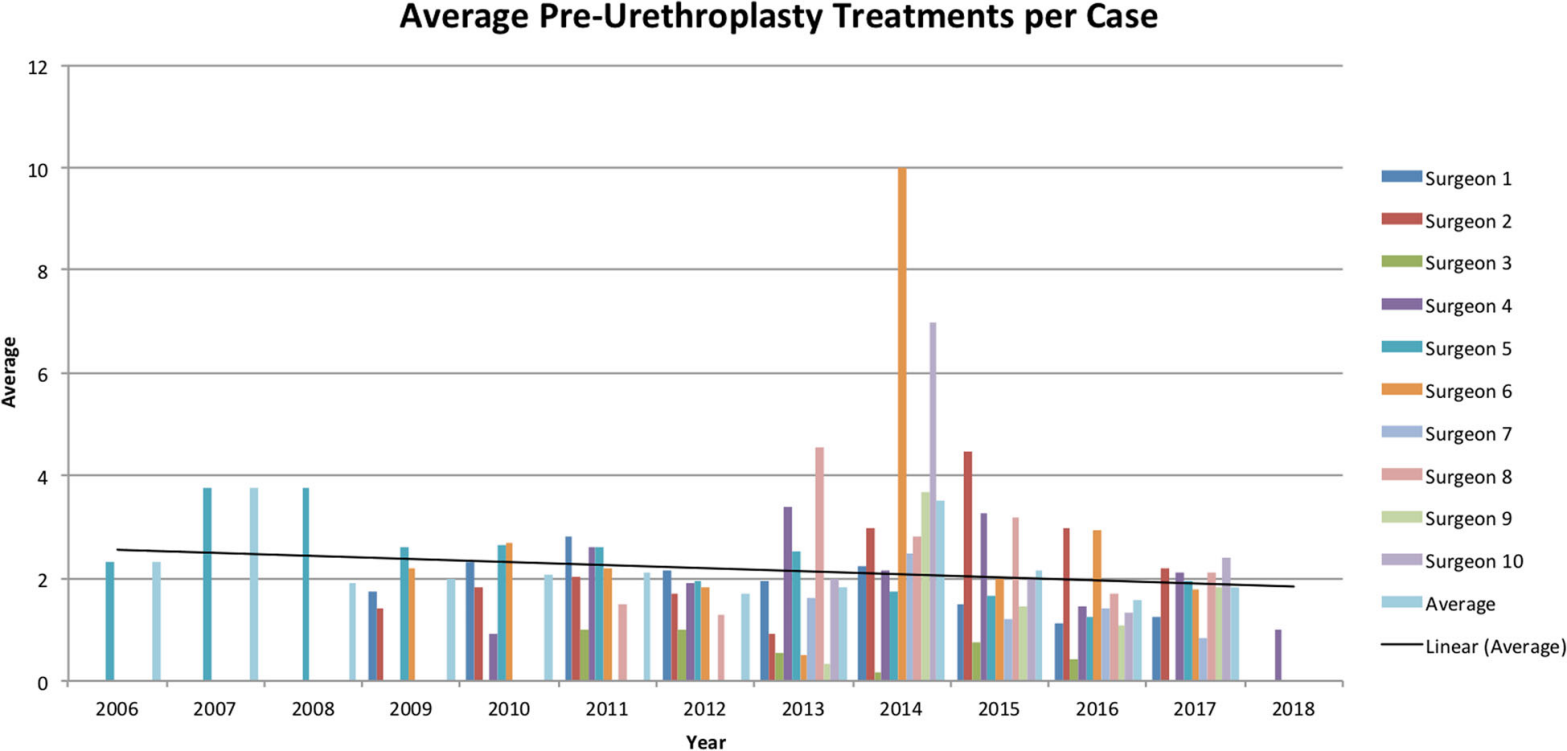
Average Pre-urethroplasty treatments per case by surgeon, per year

There was a significant decrease in the average endoscopic pre-urethroplasty treatments prior to referral between the pre-May 2016 and post-May 2016 cohorts for both overall urethroplasties (2.3 vs 1.6, *P* = 0.0012) and specifically anterior urethroplasties (2.6 vs 1.9, *P* = 0.0026). Notably however, there has been a gradual decrease in the number of pre-urethroplasty endoscopic treatments over the study period (Fig. 1). When analyzed individually, only one surgeon had an increase in average overall pre-urethroplasty endoscopic treatments of 0.09, although not statistically significant (*P* = 0.9157) (Fig. 1).

A subgroup analysis of anterior urethral strictures ≤2 cm was performed. This cohort was analyzed because the AUA USD guidelines give the option of initial endoscopic management for meatal, fossa navicularis, and bulbar urethral strictures ≤2 cm, and thereby would act as a surrogate. There were 677 patients in the pre-May 2016 subset and 218 in the post-May 2016 subset with sufficient data for analysis. There was no statistically significant difference between the pre- and post-May 2016 groups with regard to median (1 vs 1) or mean endoscopic treatments (2.04 vs 1.6, *P* = 0.1225), respectively.

## Discussion

There was a decrease in the number of endoscopic treatments of USD prior to urethroplasty in the observed period of interest. Declining endoscopic USD management is not likely to be a reflection of a solely unique influence of the guidelines as endoscopic treatment decreased over the entire study period. Interpretation of this finding however must be guarded as it seems to be a downward trend consistent with an overall change in practice patterns over the observed time period. Admittedly, previous reports have demonstrated a downward trend in the use of endoscopic management accompanied by an increase in urethroplasties [11, 12], and therefore the lack of a significant inflection point at time of guideline release in our cohort suggests the guideline is not a unique influence.

The results from this study still suggest that urethral dilation and DVIU remain the most common treatment modalities for USD management, even in areas where patients have local access to fellowship trained reconstructive surgeons. The observed number of patients with at least one endoscopic procedure in our study (69.8%) was consistent with the 65.5% rate found in a large review of Veterans Affairs data from 1999 to 2013 [13]. However, inconsistent with prior studies was our observation that significantly fewer men reported ≥2 endoscopic procedures prior to urethroplasty (37.7%) than previously reported series, where close to 70% of patients fell into this category [9, 14]. While this change may also be related to publication of the guidelines, alternative explanations include the expansion of fellowship trained reconstructive surgeons who can now support high volume urethroplasty practices, literature published prior to the release of the AUA guidelines demonstrating the cost-effective benefit of primary reconstruction over endoscopic management [15, 16], and studies emphasizing the futility of repeated endoscopic treatments [17].

Our pre-May 2016 cohort finding of an average of 2.3 pre-urethroplasty treatments for all urethroplasties and 2.6 pre-urethroplasty treatments for anterior strictures is consistent with previous studies performed during the time period [18]. Altogether, we did find a decrease in average number of pre-urethroplasty endoscopic treatments after publication of the AUA guidelines both for overall urethroplasties and anterior urethroplasties. As mentioned, in addition to the AUA guidelines, the decrease in multiple endoscopic treatments may be due to the presence of a fellowship trained reconstructive surgeon available for tertiary referral for urologists who would otherwise treat USD by endoscopic means. More reconstructive urologists are graduating from fellowships, and both younger urologists and those academically affiliated are more likely to manage strictures with urethroplasty than endoscopic treatments [19]. Admittedly, the reconstructive surgeons in our study group have been present in their geographic region for years and thus the decrease in endoscopic treatments seen after May 2016 would not be due to a new expert in the region, but rather could be explained by community urologists referring patients for urethroplasty earlier than in the past in response to the AUA guideline. We additionally note that our conclusions are limited secondary to the shorter follow up of patients after the 2016 AUA guidelines for the time period investigated in our study.

We considered urethral stricture length and stricture etiology as potential confounders of our study. However, there was no significant difference in average stricture length or in patients with lichen sclerosus for all urethroplasties between the two cohorts under investigation, which further strengthens our findings. Our results further demonstrate that the two cohort groups are fairly similar with regard to stricture etiology and therefore etiology is not likely the underlying reason for a difference in endoscopic treatments.

There are several strengths of this study that make it uniquely informative. For example, this is the largest study to look specifically at this trend with a granularity regarding patient, operative, and stricture characteristics not available in many national database sample studies since the publication of the AUA guidelines, which thereby serves to make the results more informative. A Nationwide Inpatient Sample by Buckley et al. in 2016 corroborated a similar trend to what we have shown, however our prospective database gives particular insights into these findings that can serve to tailor future research and guide more accurate conclusions [20]. The granularity of our study demonstrates that even in areas served with fellowship trained reconstructive urologists, endoscopic management of USD continues to be the dominant treatment, and thus continued research is needed to determine the barriers to earlier urethroplasty. Though, even with the new AUA guideline and trends among graduating urologists, we would be remiss not to note the significant geographic disparity in urologic reconstructive expertise that influences USD management that our study does not accurately capture [21].

Endoscopic treatments such as dilation and DVIU remain a mainstay of initial treatment for short urethral strictures. Our study demonstrates a change in practice patterns over the observed time period that progressively favor urethroplasty as an intervention with a higher long-term success rate that should be integrated earlier into USD management. The lack of a clear inflection point at the time of AUA USD guidelines release date does not necessarily infer a lack of impact, but rather suggests that guidelines continue to provide further evidence of the success of earlier referral for urethroplasty that we have shown is serving to propagate a change in contemporary practice patterns and contribute to increased utilization of urethroplasty.

## Conclusion

The number of endoscopic treatments of USD prior to referral for urethroplasty has decreased over the last decade and continues since development of the AUA male urethral stricture guideline. Despite this decline, there was no significant inflection point in the overall trend at the time of AUA publication. Further research is needed to determine further trends in the declining use of endoscopic treatment, examine long-term effects on patient outcomes with earlier referral for urethroplasty, and elucidate the barriers to earlier urethroplasty in patients with USD.

## Data Availability

The data that support the findings of this study are available from the Trauma and Urologic Reconstructive Network of Surgeons (TURNS) but restrictions apply to the availability of these data, which were used under license for the current study, and so are not publicly available. Data are however available from the authors upon reasonable request and with permission of TURNS.

## Abbreviations

AUA: American Urological Association
USD: urethral stricture disease
DVIU: direct vision internal urethrotomy
